# A retrospective cohort study in China: association between *Tripterygium* glycosides and reduced serum albumin levels in elderly rheumatoid arthritis patients

**DOI:** 10.3389/fmed.2026.1775996

**Published:** 2026-03-18

**Authors:** Huilan Guan, Hui Zhou, Fang Yuan, Meiju Zhou

**Affiliations:** 1 Department of Rheumatology and Immunology, Zhejiang Hospital, Hangzhou, Zhejiang, China; 2 Department of Emergency, Nantong Haimen People’s Hospital, Nantong, Jiangsu, China

**Keywords:** advanced age, albumin, hypoalbuminemia, rheumatoid arthritis, *Tripterygium* glycosides

## Abstract

**Introduction:**

*Tripterygium* glycosides (TGs) are widely used in rheumatoid arthritis (RA) treatment, but their potential impact on serum proteins, particularly albumin, warrants investigation. This study evaluates the effect of TGs on serum albumin levels in RA patients compared to methotrexate (MTX) or combination therapy (TGs + MTX) and identifies associated risk factors for hypoalbuminemia.

**Materials and methods:**

We conducted a retrospective study of 146 RA patients on TGs, 62 on MTX, and 54 on TGs + MTX. Serum albumin was measured before and after treatment. Logistic regression analysis was used to identify factors associated with post-treatment hypoalbuminemia.

**Results:**

Serum albumin levels did not differ significantly among the TGs, MTX, and TGs + MTX groups at baseline. Following treatment, the TGs and TGs + MTX groups exhibited significant decreases in mean serum albumin. In contrast, the MTX group showed stable albumin levels. Consequently, the incidence of hypoalbuminemia increased significantly after treatment with TGs (from 3.4 to 26.0%, *p* < 0.001) and TGs + MTX (from 0 to 18.5%, *p* < 0.001), but not with MTX alone (from 1.6 to 4.8%, *p* = 0.311). The mean changes in these serum proteins were significantly different among the three groups. Multivariable logistic regression analysis identified advanced age as a significant factor independently associated with the development of hypoalbuminemia.

**Conclusion:**

Treatment with TGs, either alone or in combination with MTX, was associated with the development of hypoalbuminemia in patients with RA. This effect was significantly associated with advanced age, suggesting a need for closer monitoring in this vulnerable population.

## Introduction

Rheumatoid arthritis (RA) is a chronic autoimmune inflammatory disease affecting synovial tissue. Common symptoms include joint discomfort, stiffness, swelling, and restricted joint movement, which can eventually lead to structural damage, deformity, and disability (1). Common systemic symptoms include morning stiffness, fatigue, fever, weight loss, and the presence of rheumatoid nodules (2). RA affects approximately 1% of the global population, with a prevalence of 1–2% in Western countries (3, 4) and around 0.62% in Eastern Mediterranean countries (5).

Current therapeutic strategies for RA primarily aim to alleviate symptoms and slow disease progression, as a complete cure remains unattainable. Early diagnosis and timely intervention are critical determinants of improved long-term outcomes (6).

Disease-modifying anti-rheumatic drugs (DMARDs) form the cornerstone of pharmacological treatment for RA, with methotrexate (MTX), leflunomide, and *Tripterygium* glycosides (TGs) being among the most commonly used agents (7, 8).

TGs, derived from the root of the *Tripterygium wilfordii* plant, which is a vine-like species native to southeastern China, and have been employed in traditional Chinese medicine for centuries. The extract contains epoxy diterpene lactones as its primary active components and exhibits potent anti-inflammatory and immunosuppressive properties (9). In modern practice, TGs are widely used to treat RA, nephrotic syndrome, and certain autoimmune conditions, demonstrating notable clinical efficacy (10, 11). Unlike traditional Chinese medicine decoctions, TGs is predominantly available in tablet form, offering convenient storage, portability, and smaller dosages compared to decoctions (12).

Despite its therapeutic benefits, TGs use is associated with notable adverse effects. Clinical observation had reported the development of hypoalbuminemia, and in a case of refractory hypoalbuminemia, whom received TGs therapy (13). The impact of TGs on serum protein homeostasis, particularly albumin, remains insufficiently studied. Therefore, this study aims to investigate the effect of TG on serum albumin levels in patients with RA and to identify potential risk factors for TGs-associated hypoalbuminemia.

## Methods

### Patients

This retrospective study enrolled patients with rheumatoid arthritis (RA) who initiated treatment with TGs at a dose of 40 mg/day and were followed up for 1 to 5 months (mean follow-up: 2 ± 1 months) were enrolled. MTX groups consisted of RA patients receiving MTX 10 mg/week who were followed up durations ranging from 0.5 to 5 months (mean follow-up: 1.9 ± 0.9 months). In TGs + MTX group, RA patients received TGs 40 mg/day and MTX 10 mg/week, who were followed up for 0.8 to 3 months (mean follow-up: 1.7 ± 0.5 months).

Exclusion criteria included: patients with dietary restrictions (e.g., vegetarian diet), malignancy, liver disease, kidney disease, gastrointestinal disorders, loss of appetite, or those who underwent surgery during the study period. Patients with poor compliance or those whose TGs or MTX dosages were adjusted during follow-up were also excluded.

All patients were diagnosed with RA according to the 2010 American College of Rheumatology/European League Against Rheumatism classification criteria for RA (14). All patients were recruited from Zhejiang Hospital between January 2020 and January 2024 and none had previously received TGs or MTX prior to enrollment.

All procedures were approved by the Ethics Committee of Zhejiang Hospital (No. 2021124k) and adhered to the tenets of the Declaration of Helsinki. Written informed consent was obtained from all participants before their inclusion in the study.

### Study design

This was a retrospective cohort study. Patients received TGs (40 mg/day), MTX (10 mg/week), or a combination of both (TGs 40 mg/day + MTX 10 mg/week), and serum biochemical parameters and disease activity were assessed before and after treatment. No dose adjustments were made during the study period.

The primary outcomes were changes in serum total protein, albumin, and globulin levels before and after treatment within each group. Secondary outcomes included comparisons of these changes across the three treatment groups.

Due to the retrospective nature of the study, it was not possible to fully control for all potential confounding factors that may influence serum albumin levels. Specifically, data on inflammatory burden (e.g., serial measurements of acute-phase reactants), concomitant infections during the follow-up period, and detailed nutritional status (e.g., dietary intake, body mass index, nutritional support) were not consistently available in the medical records and could therefore not be accounted for in the analyses.

### Data, definition, and assessment

Demographic, clinical, and laboratory data, including age, gender, disease duration, symptoms, prescriptions, laboratory data, and disease activity scores, were collected from the electronic hospital information system.

Hypoalbuminemia was defined as albumin level < 35 g/L (normal albumin level, 35–50 g/L). Elderly patient: ≥ 60 years.

Changes in disease activity were assessed using the Disease Activity Score 28 based on erythrocyte sedimentation rate (DAS28-ESR), Disease Activity Score 28 based on C-reactive protein (DAS28-CRP).

### Statistical analyses

Statistical analysis was performed using Statistical Package for Social Sciences (SPSS) software (version 22.0; IBM, USA) for Windows. All analyses were two - sided, and statistical significance was set at *p* < 0.05. All analyses were two-sided, with statistical significance set at *p* < 0.05. Continuous variables are presented as mean ± standard deviation (SD), and differences were evaluated using t-tests or Mann–Whitney-U tests. Qualitative variables are expressed as frequency (%), and chi-square tests were used to evaluate significance. Univariate logistic regression analysis identified candidate risk factors for hypoalbuminemia, followed by multivariate logistic regression on significant variables. All figures were generated using GraphPad Prism 8 software (GraphPad Software, USA).

## Results

### Patient characteristics

A total of 146 RA patients treated with TGs (mean age: 64.0 ± 9.8 years), 62 RA patients treated with MTX (mean age: 66.0 ± 5.6 years), and 54 RA patients treated with TGs + MTX (mean age: 66.0 ± 8.1 years) were included in the study.

**Figure 1:**
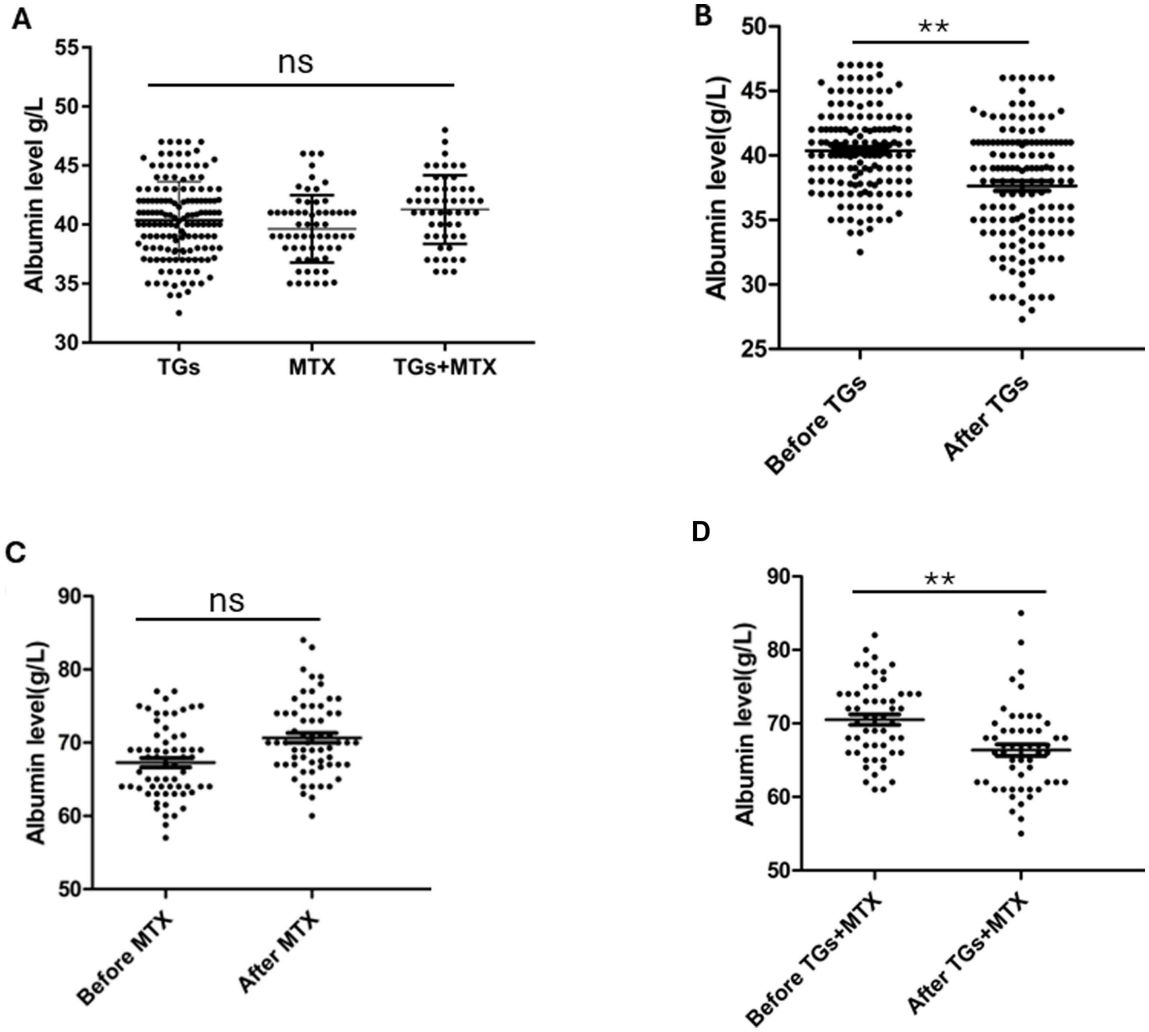
Albumin levels of TGs, MTX, and TGs + MTX group at baseline **(A)**. Albumin levels before and after TGs **(B)**, MTX **(C)**, and TGs + MTX **(D)** treatment. **p < 0.001; ns, no significance.

Table 1 presents the baseline demographic and clinical characteristics of RA patients in the TGs, MTX, and TGs + MTX groups. No significant differences were observed among the three groups with respect to age, sex, disease duration, mean follow-up duration, concomitant medications, or baseline levels of total protein, albumin, globulin, and disease activity scores (all *p* > 0.05). Figure 1A illustrates the baseline albumin levels across the three groups.

**Table 1:** Baseline characteristics of rheumatoid arthritis patients treated with TGs, MTX, and combination therapy (TGs + MTX).

	TGs group *N* = 146	MTX group *N* = 62	TGs + MTX group *n* = 54	*p* value
Age (years)	64 ± 9.8	66 ± 5.6	66 ± 8.1	0.071
Female, *n* (%)	96 (65.8%)	38 (61.3%)	41 (75.9%)	0.229
Disease duration (years)	5.8 ± 7.1	4.3 ± 5.2	5.2 ± 4.9	0.090
Mean follow-up time (months)	2 ± 1	1.9 ± 0.9	1.7 ± 0.5	0.174
DAS28-CRP	3.6 ± 1	3.4 ± 1	3.4 ± 0.4	0.416
DAS28-ESR	4.2 ± 1	4.2 ± 1	3.9 ± 0.4	0.086
Glucocorticoids, *n* (%)	66 (45.2%)	25 (40.3%)	18 (33.3%)	0.310
Iguratimod, *n* (%)	24 (16.4%)	10 (16.1%)	6 (11.1%)	0.634
Leflunomide, *n* (%)	20 (13.7%)	12 (19.4%)	9 (16.7%)	0.575
Salazosulfapyridine, *n* (%)	8 (5.5%)	3 (4.8%)	5 (9.3%)	0.546
Hydroxychloroquine, *n* (%)	22 (15.1%)	11 (17.7%)	14 (25.9%)	0.206
Tofacitinib, *n* (%)	4 (2.7%)	1 (1.5%)	4 (7.4%)	0.182
Tocilizumab, *n* (%)	3 (2.1%)	2 (3.2%)	1 (1.9%)	0.849
NSAIDs, *n* (%)	50 (34.2%)	23 (35.9%)	28 (51.9%)	0.073
Total protein (g/L)	70.4 ± 5.2	69.5 ± 4.9	70.5 ± 5.2	0.059
Albumin level (g/L)	40.4 ± 3.3	39.6 ± 3	41.3 ± 2.9	0.051
Globulin (g/L)	29.7 ± 4.9	28.4 ± 4.1	28.5 ± 5.5	0.131
Hypoalbuminemia, *n* (%)	5 (3.4%)	0	0	0.321
GPT (U/L)	21.9 ± 11.9	23.2 ± 13.0	20.3 ± 11.3	0.366
GOT (U/L)	22.3 ± 11.4	22.6 ± 11.0	22.9 ± 11.1	0.636
Total bilirubin (μmol/L)	11.7 ± 4.9	12.9 ± 5.7	10.6 ± 4.4	0.088
Serum creatine (μmol/L)	61.8 ± 20.1	51.4 ± 18.3	69.2 ± 12.3	0.966
WBC (*10^9^/L)	7.0 ± 2.3	6.8 ± 2.3	6.6 ± 1.9	0.587
HB (g/L)	125.6 ± 17.6	130.4 ± 16.5	128.7 ± 14.5	0.137
PLT (*10^9^/L)	244.5 ± 77.1	229.6 ± 73.5	236.0 ± 60.9	0.418
Proteinuria, *n* (%)	0	0	0	-

GPT, glutamic-pyruvic transaminase; GOT, glutamic oxalacetic transaminase; NSAIDs, nonsteroidal anti-inflammatory drugs; CDAI, clinical disease activity index; SDAI, simple disease activity index; DAS28-CRP, Disease Activity Score 28 based on C-reactive protein; DAS28-ESR, Disease Activity Score 28 based on erythrocyte sedimentation rate.

### Clinical outcome measures

#### TGs group

Following TGs treatment, significant decreases were observed in serum total protein (70.4 ± 5.2 vs. 66.2 ± 5.6 g/L, *p* < 0.001), albumin (40.4 ± 3.2 vs. 37.6 ± 4.5 g/L, *p* < 0.001), and globulin (29.7 ± 4.9 vs. 28.1 ± 4.8 g/L, *p* < 0.001) compared to baseline (Table 2). Accordingly, the incidence of hypoalbuminemia increased markedly from 3.4% at baseline to 26.0% post-treatment (*p* < 0.001; Table 2; Figure 1B).

**Table 2:** Characteristics of rheumatoid arthritis patients before and after treatment with TGs.

	Before TGs used *N* = 146	After TGs used *N* = 146	*p* value
Total protein (g/L)	70.4 ± 5.2	66.2 ± 6.2	< 0.001
Albumin level (g/L)	40.4 ± 3.2	37.6 ± 4.5	< 0.001
Globulin (g/L)	29.7 ± 4.9	28.1 ± 4.8	< 0.001
Hypoalbuminemia, *n* (%)	5 (3.4%)	38 (26%)	< 0.001
Total bilirubin (μmol/L)	11.7 ± 4.9	11 ± 3.9	0.178
Serum creatinine (μmol/L)	61.8 ± 20.1	62.6 ± 19.2	0.743
Proteinuria, *n* (%)	0	0	-
GPT (U/L)	21.9 ± 11.9	23 ± 11.1	0.382
GOT (U/L)	22.3 ± 11.4	24.2 ± 11.1	0.156
DAS28-CRP	3.6 ± 1	2.4 ± 0.7	< 0.001
DAS28-ESR	4.2 ± 1	3.1 ± 0.8	< 0.001
WBC (*10^9^/L)	7.0 ± 2.3	6.7 ± 2.2	*0.334*
Hb (g/L)	125.6 ± 17.6	128.3 ± 14.9	*0.156*
PLT (*10^9^/L)	244.5 ± 77.1	236.0 ± 51.8	*0.268*

GPT, glutamic-pyruvic transaminase; GOT, glutamic oxalacetic transaminase; NSAIDs, nonsteroidal anti-inflammatory drugs; CDAI, clinical disease activity index; SDAI, simple disease activity index; DAS28-CRP, Disease Activity Score 28 based on C-reactive protein; DAS28-ESR, Disease Activity Score 28 based on erythrocyte sedimentation rate.

Simultaneously, disease activity measures, DAS28-CRP (3.6 ± 1.0 vs. 2.4 ± 0.7, *p* < 0.001), and DAS28-ESR (4.2 ± 1.0 vs. 3.1 ± 0.8, *p* < 0.001), significantly decreased after TGs administration compared to pre-treatment levels. There were no significant differences in the serum items before and after TGs used in glutamic pyruvic transaminase (GPT), glutamic oxalacetic transaminase (GOT), total bilirubin, proteinuria, proteinuria, and serum creatinine (sCr) in the TGs group (Table 2).

#### MTX group

In the MTX group, disease activity also improved significantly, with DAS28-CRP decreasing from 3.7 ± 1.0 to 2.5 ± 0.6 (*p* < 0.001) and DAS28-ESR from 4.2 ± 1.0 to 3.3 ± 0.8 (*p* < 0.001). However, no significant differences were detected in serum total protein, albumin, globulin, or the incidence of hypoalbuminemia following MTX treatment (all *p* > 0.05; Table 3; Figure 1C). Liver and renal function parameters, as well as proteinuria, remained stable (all *p* > 0.05; Table 3).

**Table 3:** Characteristics of rheumatoid arthritis patients before and after treatment with MTX.

	Before MTX *N* = 62	After MTX *N* = 62	*p* value
Total protein (g/L)	69.5 ± 4.9	70.7 ± 5.1	0.131
Albumin level (g/L)	39.6 ± 3	40.4 ± 4.2	0.142
Globulin (g/L)	28.4 ± 4.1	29.7 ± 4.9	0.112
Hypoalbuminemia, *n* (%)	1 (1.6%)	3 (4.8%)	0.311
Total bilirubin (μmol/L)	11 ± 3.9	11.3 ± 4.3	0.089
Serum creatinine (μmol/L)	62.6 ± 19.2	61.9 ± 16.9	0.875
Proteinuria, *n* (%)	0	0	-
GPT (U/L)	23 ± 11.1	28.5 ± 11.4	0.223
GOT (U/L)	24.2 ± 11.1	26.3 ± 11.7	0.089
DAS28-CRP	3.7 ± 1.0	2.5 ± 0.6	< 0.001
DAS28-ESR	4.2 ± 1.0	3.3 ± 0.8	< 0.001
WBC (*10^9^/L)	6.8 ± 2.3	6.7 ± 2.0	0.673
Hb (g/L)	130.4 ± 16.3	132.5 ± 14.8	0.465
PLT (*10^9^/L)	229.6 ± 73.5	230.2 ± 41.8	0.956

GPT, glutamic-pyruvic transaminase; GOT, glutamic oxalacetic transaminase; NSAIDs, nonsteroidal anti-inflammatory drugs; CDAI, clinical disease activity index; SDAI, simple disease activity index; DAS28-CRP, Disease Activity Score 28 based on C-reactive protein; DAS28-ESR, Disease Activity Score 28 based on erythrocyte sedimentation rate.

#### TGs + MTX group

Patients receiving combination therapy (TGs + MTX) exhibited significant reductions in serum total protein (70.5 ± 5.2 vs. 66.4 ± 5.8, *p* < 0.001), albumin (41.3 ± 2.9 vs. 38.3 ± 3.7, *p* < 0.001), and globulin (28.5 ± 5.5, 27.4 ± 4.5, *p* = 0.023) compared to baseline. The prevalence of hypoalbuminemia increased significantly from 0% at baseline to 18.5% post-treatment (*p* < 0.001).

Disease activity improved substantially, as indicated by decreased DAS28-CRP (3.2 ± 0.4 vs. 2.3 ± 0.5, *p* < 0.001) and DAS28-ESR (3.8 ± 0.4 vs. 2.9 ± 0.6, *p* < 0.001). No significant changes were noted in liver or renal function markers or proteinuria following combination therapy (all *p* > 0.05; Table 4; Figure 1D).

**Table 4:** Characteristics of rheumatoid arthritis patients before and after treatment with TGs and MTX combination therapy.

	Before TGs + MTX *N* = 146	After TGs + MTX *N* = 146	*p* value
Total protein (g/L)	70.5 ± 5.2	66.4 ± 5.8	<0.001
Albumin level (g/L)	41.3 ± 2.9	38.3 ± 3.7	<0.001
Globulin (g/L)	28.5 ± 5.5	27.4 ± 4.5	0.023
Hypoalbuminemia, *n* (%)	0	10	<0.001
Total bilirubin (μmol/L)	10.6 ± 4.4	9.9 ± 3.8	0.465
Serum creatinine (μmol/L)	61.2 ± 16.3	62.1 ± 15.1	0.767
Proteinuria, *n* (%)	0	0	-
GPT (U/L)	20.3 ± 11.3	19.8 ± 10.7	0.906
GOT (U/L)	22.9 ± 10.1	21.5 ± 8.7	0.362
DAS28-CRP	3.2 ± 0.4	2.3 ± 0.5	<0.001
DAS28-ESR	3.8 ± 0.4	2.9 ± 0.6	<0.001
White blood cell (*10^9^/L)	6.6 ± 1.9	7.5 ± 8.0	0.746
Hemoglobin (g/L)	128.7 ± 14.5	128.1 ± 12.0	0.769
Platelet (*10^9^/L)	236.0 ± 60.9	219.0 ± 62.2	0.109

GPT, glutamic-pyruvic transaminase; GOT, glutamic oxalacetic transaminase; NSAIDs, nonsteroidal anti-inflammatory drugs; CDAI, clinical disease activity index; SDAI, simple disease activity index; DAS28-CRP, Disease Activity Score 28 based on C-reactive protein; DAS28-ESR, Disease Activity Score 28 based on erythrocyte sedimentation rate.

### Characteristics associated with hypoalbuminemia in the TGs group

Table 5 compares the characteristics of RA patients with and without hypoalbuminemia after TGs treatment in the TGs group. Total of 146 RA patients, 38 (26%) developed hypoalbuminemia after TGs treatment. Compared to the RA patients who did not develop hypoalbuminemia, patients who developed hypoalbuminemia were older (70 ± 12.2 vs. 62 ± 7.9, *p* < 0.001), with more patients aged ≥ 70 years (47.4% vs. 13.9%, *p* < 0.001) and ≥80 years (28.9% versus 3.7%, *p* < 0.001) than those in the group with normal albumin levels after TGs intervention.

**Table 5:** Characteristics of RA patients with and without hypoalbuminemia after TGs treatment.

	With hypoalbuminemia *N* = 38	Without hypoalbuminemia *N* = 108	*p* value
Female (*n*, %)	25 (65.8%)	71 (65.7%)	0.853
Age (years)	70 ± 12.2	62 ± 7.9	< 0.001
Age ≥ 80 (*n*, %)	11 (28.9%)	4 (3.7%)	< 0.001
Age 70–80 (*n*, %)	18 (47.4%)	15 (13.9%)	< 0.001
Age 60–70 (*n*, %)	29 (76.3%)	64 (59.3%)	0.060
Glucocorticoids, *n* (%)	15 (39.5%)	51 (47.2%)	0.524
Iguratimod, *n* (%)	8 (21.1%)	16 (14.8%)	0.525
Leflunomide, *n* (%)	4 (10.5%)	16 (14.8%)	0.713
Salazosulfapyridine, *n* (%)	3 (7.9%)	5 (4.6%)	0.735
Hydroxychloroquine, *n* (%)	6 (15.8%)	16 (14.8%)	0.912
Tofacitinib, *n* (%)	1 (2.6%)	3 (2.8%)	0.586
Tocilizumab, *n* (%)	1 (2.6%)	2 (1.9%)	0.772
NSAIDs (*n*, %)	14 (36.8%)	36 (33.3%)	0.891

NSAIDs, nonsteroidal anti-inflammatory drugs.

### Comparison of changes across groups

**Figure 2:**
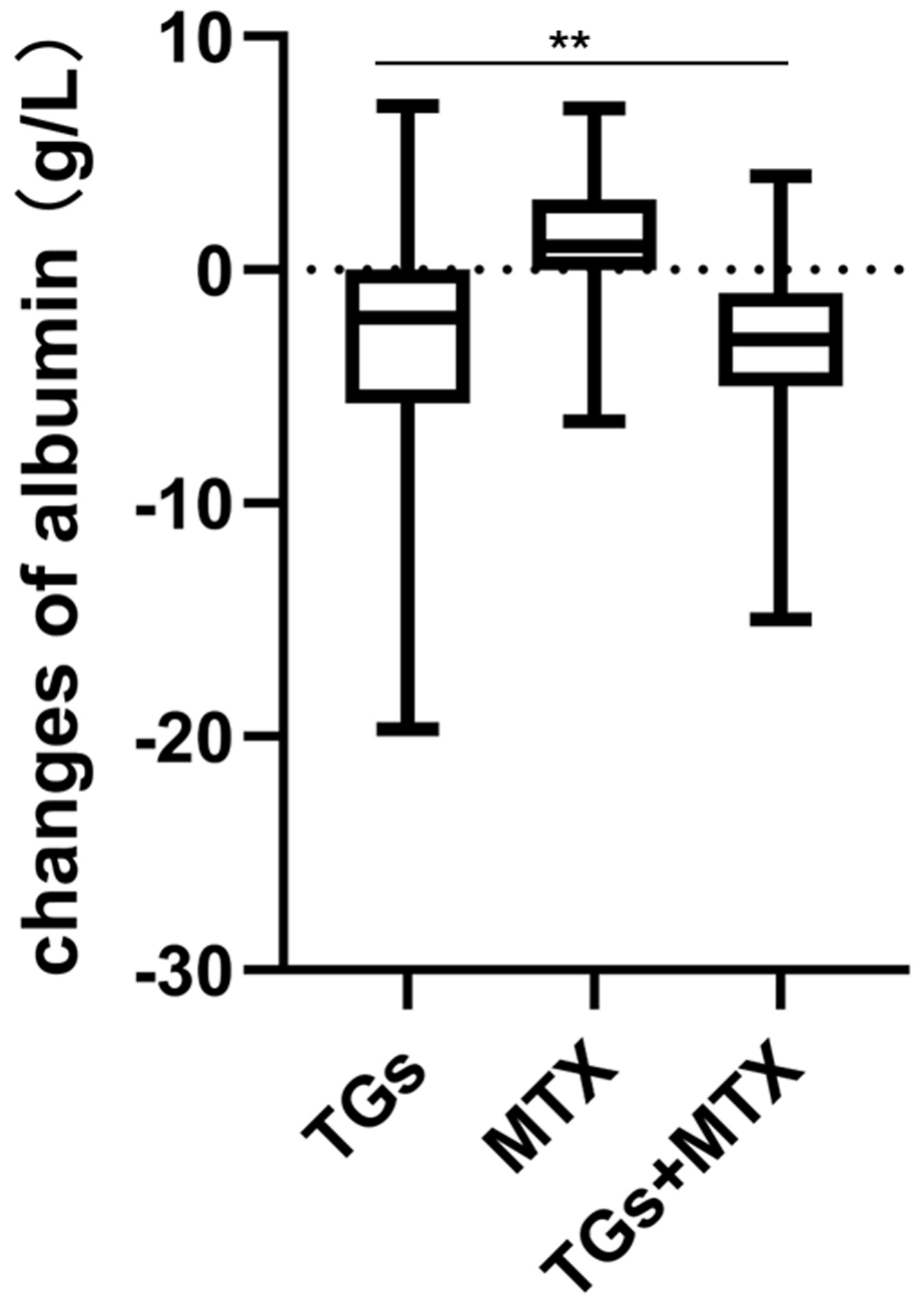
Mean changes in serum albumin from baseline to follow-up in RA patients receiving TGs, MTX, or combination therapy. **p < 0.001; ns, no significance.

Significant differences were observed in the mean changes of albumin (−2.7 ± 4.6 vs. 1.4 ± 2.9 vs. − 3.1 ± 3.3, *p* < 0.001) among the TGs, MTX, and TGs + MTX groups from baseline to the follow-up period (Figure 2). In contrast, no significant differences were found in the mean changes of disease activity among the three groups during the same period.

And we have evaluated correlations between changes in C-reactive protein and serum albumin, no significant correlations were observed.

### Risk factors for hypoalbuminemia

To identify independent risk factors for hypoalbuminemia in RA patients receiving TGs treatment, univariate and multivariate logistic regression analyses were performed. In the univariate analysis, the following variables were examined: age, sex, disease duration, baseline disease activity (DAS28-ESR and DAS28-CRP), baseline albumin level, and concomitant use of glucocorticoids, DMARDs, or NSAIDs. Among these, age (*p* < 0.001) showed significant associations with post-treatment hypoalbuminemia and were entered into the multivariate model.

Logistic regression analysis suggested age (OR = 1.089, 95% CI: 1.044–1.136, *p* < 0.001) as a risk factor for hypoalbuminemia in patients with RA receiving TGs treatment. Specifically, advanced age significantly correlated with the risk of developing hypoalbuminemia in patients with RA. For instance, patients with RA aged ≥ 80 years (OR = 10.593, 95% CI: 3.127–35.885, *p* < 0.001) and ≥ 70 years (OR = 5.580, 95% CI: 2.413–12.904, *p* < 0.001) exhibited a significantly high risk of hypoalbuminemia after TGs intervention.

## Discussion

RA is a group of rheumatic and autoimmune diseases characterized by immune system disorders. In RA, the hyperactive immune system erroneously attacks bodily tissues and organs, leading to inflammation, tissue damage, and functional impairment. Treatment strategies for RA focus on modulating immune responses, reducing inflammation, alleviating symptoms, and specifically targeting damaged organs (15). Although several biological therapies are available, traditional DMARDs remain the primary treatment option for RA.

*Tripterygium*, a genus in the family Celastraceae, is widely distributed throughout East Asia and includes species such as *T. wilfordii, T. hypoglaucum, and T. regelii*. These plants are primarily lianas and shrubs (16). In recent decades, *Tripterygium* has garnered significant attention from experts due to its unique medical value, important pharmacological effects, and complex chemical compositions. The polycoride extracted from *T. wilfordii* is considered one of the most effective “Chinese herbal hormones” and is extensively used in managing RA (17), primary Sjogren’s syndrome (18) and psoriasis (19). In China, TGs are available in tablet form and have been shown to effectively improve symptoms and quality of life in patients with autoimmune diseases (15).

In this retrospective study, we analyzed 146 RA patients treated with TGs, 62 treated with MTX, and 54 treated with combination therapy (TGs + MTX). Our findings demonstrate that TGs, either alone or in combination with MTX, significantly reduced disease activity in RA patients, confirming its therapeutic efficacy. However, this clinical benefit was accompanied by a notable decrease in serum albumin levels and a marked increase in the incidence of hypoalbuminemia. Importantly, no significant changes were observed in liver function indices (GPT, GOT, total bilirubin), renal function markers (serum creatinine), or proteinuria following TGs treatment, suggesting that the observed hypoalbuminemia was not attributable to overt hepatorenal toxicity.

Despite its therapeutic benefits, TGs are associated with a range of adverse effects, including gastrointestinal, reproductive, hepatic, renal, hematologic, and cutaneous toxicity (20, 21). A previous study involving 2,347 patients reported that over 30% experienced adverse reactions, predominantly occurring within the first 3 months of treatment (22). While some patients developed tolerance over time, and most adverse effects resolved upon drug discontinuation.

Human serum albumin is a key non-glycosylated plasma carrier protein with various functions. It interacts with various ligands, including exogenous drugs, playing a critical role in compound transport, ligand binding, distribution, and metabolism. Synthesized by liver parenchymal cells, serum albumin has a half-life of 15–18 days in the plasma but is not stored in the liver (23). Serum albumin levels can be influenced by dietary factors, liver function, kidney function, and other factors. Given its numerous properties, including binding and regulation of metabolism and drug dosage, albumin is considered one of the most significant indicators in the human body (24, 25).

Serum albumin, synthesized exclusively by hepatocytes, is the most abundant plasma protein and plays critical roles in maintaining oncotic pressure, transporting endogenous and exogenous ligands, and modulating drug pharmacokinetics (23). Hypoalbuminemia has been associated with disease severity, increased infection risk, malnutrition, thrombosis, edema, impaired wound healing, and adverse clinical outcomes (24–26). Despite its clinical significance, serum albumin is often overlooked in routine practice, and the potential impact of TGs on albumin homeostasis has not been previously investigated.

Hypoalbuminemia has been closely associated with disease severity and adverse outcomes (26). Although serum albumin is a nonspecific index that may be easily overlooked in clinical practice, long-term hypoalbuminemia can lead to complications, such as infection, malnutrition, thrombosis, edema, poor wound healing, and heart failure. To our knowledge, this study is the first to report TGs-induced hypoalbuminemia in RA patients. The specific mechanism underlying this phenomenon remains unclear. However, the specific mechanism underlying TGs-induced hypoalbuminemia remains unknown.

Zhou et al. (25) examined serum biochemical parameters, liver histopathological changes, the expression of liver inflammatory factors, and NLRP3 inflammasome activation after triptolide administration. They suggested that triptolide activated the TLR4-Myd88-NF-κB pathway and induced oxidative stress, potentially participating in NLRP3 inflammasome activation, leading to liver injury and hypoalbuminemia. However, in the current study, serum liver function indexes remained normal before and after TGs administration, indicating that TGs might affect albumin metabolism through alternative pathways or liver pathologies.

In another study, Yang et al. (27) used network toxicology and molecular docking to show that *Tripterygium wilfordii* Hook. F might have induced kidney injury through mediating inflammation via the PI3K-Akt/HIF-1/TNF signaling pathway. It progresses to renal inflammation and mediates hypoxia via the HIF-1 signaling pathway to accelerate inflammatory processes. These processes may increase malondialdehyde and urea nitrogen levels, causing oxidative stress and structural damage to the kidneys, thereby increasing the filtration rate of albumin in glomeruli (23, 27). However, in the current study, no significant difference was observed in the serum creatine kinase levels and proteinuria before and after TGs administration.

Logistic regression analysis also indicated that advanced age was correlated with hypoalbuminemia in RA patients treated with TGs, possibly due to differences in TGs metabolism and albumin production in older patients compared to younger patients. However, the precise mechanism underlying the TGs-induced decline in human albumin levels remains unclear.

In clinical practice, most patients with hypoalbuminemia are treated with intravenous human blood albumin, however, the underlying cause has rarely been explored. TGs-induced hypoalbuminemia can be challenging to manage and may necessitate TGs discontinuation. Therefore, this study demonstrated that regular monitoring of serum albumin levels in patients treated with TGs is recommended for early detection and treatment, thereby preventing serious complications. We first found TGs induced hypoalbuminemia, and the phenomenon was correlated with elderly age, but the underlying mechanisms were not explored. In clinical, discontinued TGs, albumin level can gradually restore to normal level, and which is still under study.

Several limitations should be considered when interpreting our findings. First, the retrospective, single-center design introduces inherent selection bias and limits generalizability. Despite statistical adjustments, residual confounding from unmeasured variables, such as nutritional status, concomitant infections, and inflammatory burdens cannot be excluded. Second, the sample size was modest and uneven across groups, which may have reduced statistical power for subgroup analyses. Third, the relatively short follow-up period (mean 1.7–2.0 months) precluded assessment of long-term outcomes, including persistence of hypoalbuminemia after TGs discontinuation and its association with clinical complications (e.g., infections, edema, cardiovascular events). Fourth, the absence of mechanistic investigations leaves the biological pathways underlying TGs-induced hypoalbuminemia speculative. Potential mechanisms, including hepatotoxicity, renal protein loss, gastrointestinal malabsorption, or direct inhibition of albumin synthesis, remain unverified without supporting laboratory or histopathological data.

More detailed protein-level investigations, such as serum protein electrophoresis and subsequent protein-specific analyses (Western blotting), would help clarify the biological basis of the observed hypoalbuminemia. Discussion of markers like prealbumin, immunoglobulin subclasses, or acute-phase protein patterns, would have strengthened causal inference.

Despite these limitations, this study provides the first evidence linking TGs therapy to hypoalbuminemia in RA patients and identifies advanced age as a key risk factor. Prospective, multicenter studies with larger cohorts, extended follow-up, standardized confounder adjustment, and integrated mechanistic evaluations are urgently needed to validate these findings and inform evidence-based monitoring strategies. Future research should prioritize elderly RA populations to clarify age-specific risks and optimize therapeutic algorithms.

## Conclusion

This retrospective cohort study examined hypoalbuminemia in patients with RA treated with TGs. The results indicate that TGs therapy is associated with the development of hypoalbuminemia in RA patients, particularly in those of advanced age. And on a global scale, our team was the first to discover this phenomenon.

## Data Availability

The datasets presented in this study can be found in online repositories. The names of the repository/repositories and accession number(s) can be found in the article/supplementary material.
